# A separable convolutional neural network-based fast recognition method for AR-P300

**DOI:** 10.3389/fnhum.2022.986928

**Published:** 2022-10-19

**Authors:** Chunzhao He, Yulin Du, Xincan Zhao

**Affiliations:** 3D Immersive Computing and Display Lab, School of Computer and Artificial Intelligence, Zhengzhou University, Zhengzhou, China

**Keywords:** convolutional neural network (CNN), augmented reality (AR), brain-computer interfaces (BCI), P300, single extraction

## Abstract

Augmented reality-based brain–computer interface (AR–BCI) has a low signal-to-noise ratio (SNR) and high real-time requirements. Classical machine learning algorithms that improve the recognition accuracy through multiple averaging significantly affect the information transfer rate (ITR) of the AR–SSVEP system. In this study, a fast recognition method based on a separable convolutional neural network (SepCNN) was developed for an AR-based P300 component (AR–P300). SepCNN achieved single extraction of AR–P300 features and improved the recognition speed. A nine-target AR–P300 single-stimulus paradigm was designed to be administered with AR holographic glasses to verify the effectiveness of SepCNN. Compared with four classical algorithms, SepCNN significantly improved the average target recognition accuracy (81.1%) and information transmission rate (57.90 bits/min) of AR–P300 single extraction. SepCNN with single extraction also attained better results than classical algorithms with multiple averaging.

## Introduction

The study of the brain–computer interface (BCI) currently centers on brain–computer interaction (Xu et al., 2021). Augmented reality (AR) can be used to enhance brain–computer interaction, and in the future, the integration of AR wearable devices and BCI will be possible. AR-based steady-state visual evoked potential (SSVEP) BCI is receiving increased attention from researchers (Lenhardt and Ritter, 2010; Kerous and Liarokapis, 2017; Si-Mohammed et al., 2018; Zhao et al., 2021). However, compared with a computer screen (CS) (Lenhardt and Ritter, 2010) or a virtual reality (VR) device (Rohani and Puthusserypady, 2015), the quality of AR stimuli superimposed on real scenes is susceptible to environmental influences, making target recognition difficult. Algorithms such as linear discriminant analysis (LDA) (Bostanov, 2004) and support vector machine (SVM) (Kaper et al., 2004) are often used for the identification of AR–BCI. Mohammed et al. designed an AR–SSVEP paradigm for controlling robots. The accuracy rate of LDA was 82% under a stimulus lasting 7 s (Si-Mohammed et al., 2018). However, longer stimulation times reduce system classification speed. In the following study (Zhao et al., 2021), the AR–SSVEP stimulation time was shortened to 1 s using a deep learning algorithm, which significantly improved the information transfer rate (ITR) of the AR–SSVEP system, and the accuracy was still reasonable at 80%. Compared with the SSVEP, the P300-based BCI (P300–BCI) has a large command set (Jin et al., 2012) and induces little visual fatigue (Farwell and Donchin, 1988; Sellers and Donchin, 2006; Nijboer et al., 2008). The combination of AR and P300–BCI may have the potential to significantly improve the degrees of freedom and practicability of AR–BCI.

Kouji et al. designed a four-target AR–P300 single-stimulus paradigm to control home appliances, and the recognition accuracy of the LDA algorithm was 82.7% after 15 averages (Kouji et al., 2011). Ahn et al. designed a seven-target P300 single-stimulus paradigm for drone control and conducted experiments in VR and AR environments. The recognition accuracy of the LDA algorithm after 20 averages reached 90.88% in the VR environment and 88.53% in the AR environment (Kim et al., 2021). Although the LDA algorithms developed in these two studies achieved good recognition accuracies, the use of multiple averaging seriously reduced their classification speed.

Deep learning algorithms are expected to improve the performance of AR–P300 single extraction. Cecotti et al. used a convolutional neural network (CNN) consisting of two convolutional layers and a fully connected layer to recognize the P300 oddball paradigm for the first time (Cecotti and Graser, 2011). Manor and Geva proposed an architecture including three convolutional layers and two fully connected layers for classification in Rapid Serial Visual Presentation (RSVP), and the area under the curve (AUC) in single extraction was 0.77 (Manor and Geva, 2015). Inspired by separable convolution (Chollet, 2017), Lawhern et al. proposed a compact CNN architecture consisting of a standard convolution layer, a separable convolution layer, and a fully connected layer, achieving an AUC of 0.92 in single extraction in the P300 oddball paradigm (Lawhern et al., 2018). Subsequent studies gradually reduced the model complexity and proposed a simpler single-layer CNN structure (Shan et al., 2018; Alvarado-González et al., 2021). The deep learning algorithm has achieved good performance in the classification of CS–P300. Whether a neural network can be designed to effectively extract AR–P300 features with a single extraction in order to construct a fast AR–BCI system remains to be studied. In this study, a fast recognition method for AR–P300 was developed based on a separable convolutional neural network (SepCNN).

The layout of the paper is as follows. In section “Materials and methods,” the experiments, the methods used for data preprocessing, and the structure of SepCNN are described. In section “Comparison of single extraction results obtained by SepCNN and classical algorithms,” the recognition results of SepCNN and classical algorithms for AR–P300 single extraction are presented to verify the effectiveness of SepCNN. Section “Comparison results of SepCNN in single extraction and classic algorithm in multiple averaging” compares SepCNN with classical algorithms to further verify the performance advantages of SepCNN. Section “Discussion” discusses the influencing factors of SepCNN and the performance difference from standard CNN.

## Materials and methods

### Participants and experimental environment

**FIGURE 1:**
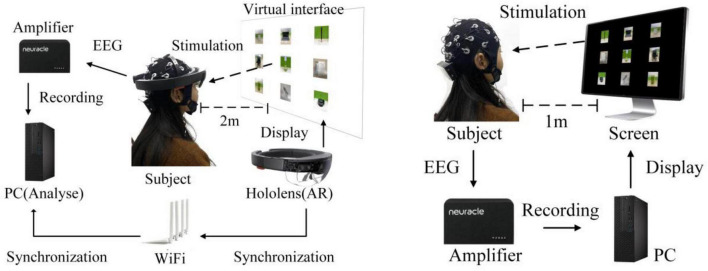
Experimental environment in panels **(A)** AR–P300 and **(B)** CS–P300.

**FIGURE 2:**
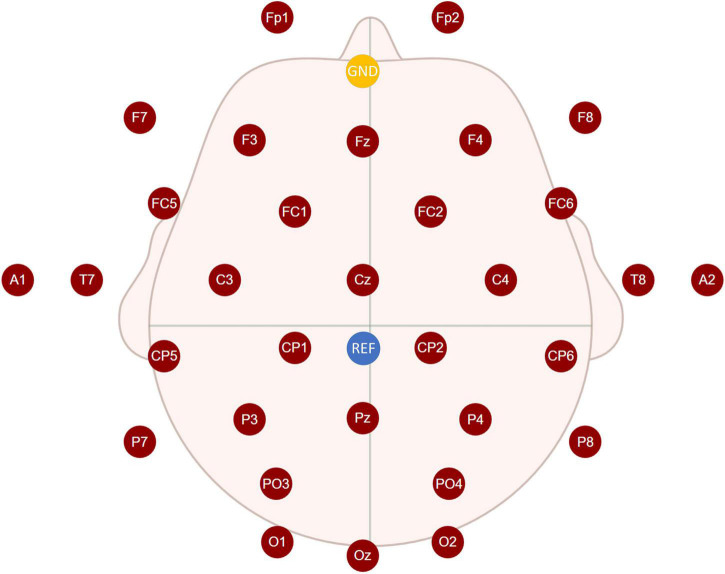
Locations of all electrodes.

Fifteen healthy participants (two females, 13 males; aged 23 ± 2.6 years) with normal or corrected vision volunteered to participate in the experiment. All participants read and signed informed consent forms before the experiment. Each participant had to perform the same experiment in both an AR environment, *via* HoloLens glasses (see Figure 1A), and a CS environment, *via* a CS display (see Figure 1B). The hardware parameters of the CS and HoloLens glasses are provided in Table 1. Electroencephalogram (EEG) signals were recorded in a quiet, dark room to reduce interference from strong light. EEGs were recorded at a 1,000-Hz sampling rate from 32 electrodes placed at the standard positions of the international 10–20 system. Figure 2 shows the locations of all electrodes, with the ground wire in yellow and the reference electrode in blue. A Neuracle amplifier was used for amplification and analog-to-digital conversion of the EEG signals. Its details were shown in Table 2. The impedance of all electrodes was less than 10 kΩ. The CS-P300 and AR-P300 were scheduled for the first and second day respectively to avoid excessive fatigue of the participants. Before the start of both experiments, participants were asked to complete two or more pre-experiments to understand the experimental process and adapt to the BCI.

**TABLE 1:** Hardware parameters of HoloLens and personal computer (PC).

Environment	CPU	GPU	Memory (RAM)
AR	Intel Atom x5–Z8100 @ 1.04 GHz	–	2 GB
PC	Intel Core i5–7500 @ 3.40 GHz	Quadro K2200 (4 GB)	16 GB

**TABLE 2:** Details of electroencephalogram (EEG) amplifier.

Item	Detail
Company Name	Neuracle
Company Address	Changzhou City, Jiangsu Province, China
Product Name	NeuSen W series wireless EEG acquisition system

### Experimental setup

**FIGURE 3:**
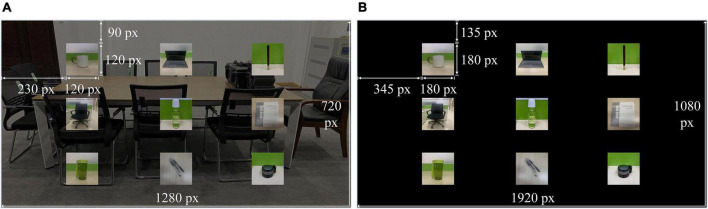
Distribution of P300 stimuli in panels **(A)** AR–P300 and **(B)** CS–P300.

The AR–P300 stimulation interface was designed using the HelmetSceneGraph (HSG) AR rendering engine independently developed by our laboratory and presented on HoloLens (resolution 1,280 × 720, refresh rate 60 Hz), with a transparent background. The size of each stimulus image (a common object) was 120 × 120 pixels, the interface was 1,280 × 720 pixels, and the visual distance was 2 m, as shown in Figure 3A. The CS–P300 visual stimuli were presented on a 27-inch monitor (resolution 1,920 × 1,080, refresh rate 60 Hz), the visual stimulation interface was achieved through the MATLAB + Psychtoolbox with a black background, and the visual distance was 1 m, as shown in Figure 3B.

**FIGURE 4:**
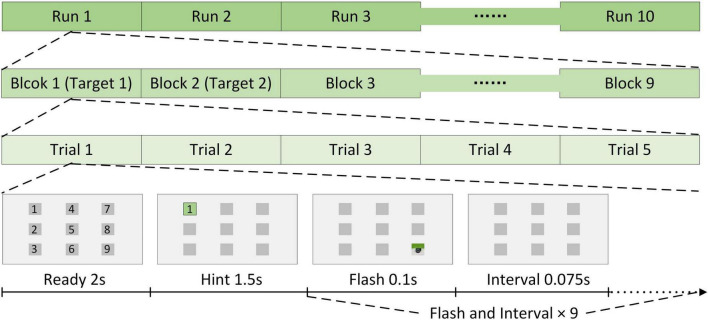
Stimulation method.

The stimulation method was the same for AR–P300 and CS–P300 (see Figure 4), and each participant had to perform 10 identical runs in each experimental environment. Each run contained nine different blocks corresponding to nine different stimuli. Each block contained five repeated trials. In a trial, 2 s was used for preparation and 1.5 s was used for target lock. Next, nine stimuli flashed once in random order. Existing studies have shown that a shorter inter-stimulus interval (ISI) can produce higher classification accuracy and ITR (Sellers et al., 2006). We referred to this study and setted an ISI of 175 ms to ensure that the classification results and ITR of each algorithm were close to optimal. As a result, for each flash, the duration was 100 ms and the interval was 75 ms. In each run, the participants were asked to follow the hints to focus on nine target stimuli from top to bottom and left to right, and only one stimulus was focused per block. Due to the different preparation times for the two experiments, the total durations of the AR–P300 and CS–P300 experiments were about 40 and 30 min, respectively. In the AR-BCI experiment, participants tended to rest longer because of the Hololens squeeze on the forehead and neck. In addition, most participants took longer to initiate the experimental procedure because of the unskilled control of the Hololens.

### Preprocessing

**FIGURE 5:**
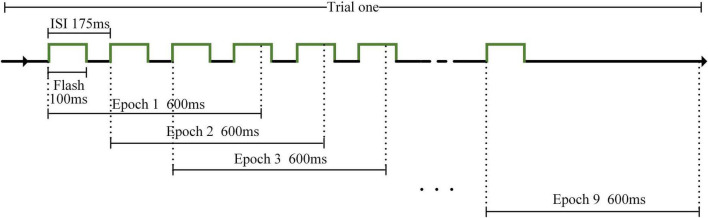
Electroencephalogram (EEG) division of AR–P300.

Electroencephalograms of channels A1 and A2 were deleted, and the reference electrode was REF near Cz (see Figure 2). A bandpass filter with cut-off frequencies of 0.1 and 12 Hz was applied to the EEGs. Each trial generated nine epochs for analysis, as shown in Figure 5. The algorithms had to intercept an epoch of 600 ms after each flash for analysis. Therefore, when the last stimulus started flashing, the experiment ended in 600 ms instead of 175 m. Each participant generated target and non-target stimulus data sets, which contained 450 and 3,600 data points, respectively, in one experimental environment. The dimension of each data point is 600 × 30 (where the dimensions are expressed as data length × number of channels) and downsampled to 200 × 30 to input SepCNN and classical machine learning algorithms.

### Traditional recognition method

Traditional algorithms were implemented in MatLab. We chose four classical methods as baselines. LDA projects EEGs from a high-dimensional space to a low-dimensional space and classifies samples by learning an optimal hyperplane (Guger et al., 2009). Bayesian linear discriminant analysis (BLDA) is an extension of LDA and prevents the overfitting of high-dimensional EEG data through regularization (Hoffmann et al., 2008). SVM transforms the EEG into a high-dimensional space through nonlinear mapping and maximizes the inter-class distance by constructing a hyperplane to improve recognition accuracy (Vapnik, 1998). The linear kernel was used for the SVM kernel function in this paper and the hyper-parameter C was 1. Stepwise linear discriminant analysis (SWLDA) reduces data dimensionality by optimizing EEG local features and comprehensively tests the current and previous EEG feature variables to retain the significant features that can indicate the sample (Krusienski et al., 2006). In this paper, the maximum *p*-value was 0.1 for predictors to add, the minimum *p*-value was 0.15 for predictors to remove, and the maximum number of steps to perform was 60.

### Separable convolutional neural network and standard convolutional neural network

**FIGURE 6:**
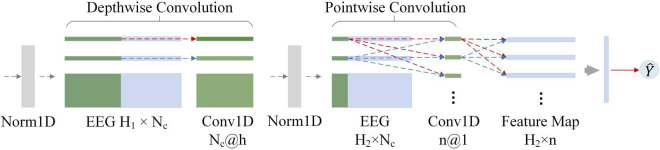
Separable convolutional neural network (SepCNN) structure. H_1_ is the length of the input data, N_c_ is the number of channels, h is the size of the depthwise convolution kernel, H_2_ is the input data length of the second layer, and n is the number of pointwise convolutions.

The neural network was implemented by Python and PyTorch. Figure 6 shows the architecture of the SepCNN model for P300 target recognition. Table 3 details the model parameters for the participant-independent training (within-participant). The standard CNN structure was used for further comparison with SepCNN. In this manuscript, for the within-participant training, the standard CNN consisted of a normalization layer, a one-dimensional (1D) standard convolutional layer, a Tanh layer, a fully connected layer and a sigmoid layer. In a standard convolution layer, each input was convolved with all convolution kernels separately, which was similar to the pointwise convolution (see Figure 6). For a fair comparison, the hyperparameter settings of standard CNN were almost the same as those of SepCNN. The hyperparameter of a 1D standard convolutional layer included InChannels (30), OutChannels (4), Size (10), Stride (6), and Padding (4).

**TABLE 3:** Separable convolutional neural network (SepCNN) architecture for within-participant recognition.

Layer	Kernel	Size	Options	Output
Input		200 × 30		
BatchNorm1D				200 × 30
DepthConv1D	30	10	Stride = 6, Padding = 4	34 × 30
BatchNorm1D				34 × 30
PointConv1D	4	1	Stride = 1, Padding = 0	34 × 4
Activation			Tanh	34 × 4
Flatten				136
Activation			Sigmoid	1

Unlike standard convolution, separable convolution processes the channel and time dimensions separately via depthwise convolution and pointwise convolution. The number of depthwise convolution kernels is equal to the number of channels. Each kernel processes each channel independently to learn channel features in different time dimensions. The operation of pointwise convolution is similar to that of standard convolution, but the kernel size has to be one. Pointwise convolution effectively extracts the features of different channels in the same time dimension through the weighted combination of the input feature map in the channel dimension. Therefore, pointwise convolution increases the number of non-linear layers without significantly increasing the parameters, thereby expanding the possibilities of the model. In the AR-P300, due to the different adaptability of the participants to the binocular AR environment, there were large differences in their visual perception and EEG. Therefore, a normalization layer was added before depthwise and pointwise convolutional layer to concentrate the sample features, which can reduce the adverse effects of abnormal samples. A tanh activation function is used in front of the fully connected layer to enhance the feature difference between target and non-target stimuli. Finally, the sigmoid activation function is used to predict the probability of a stimulus being the target stimulus.

In addition, single-layer one-dimensional (1D) separable convolution requires fewer parameters than standard 1D convolution, and its computational cost is expressed as follows:


$$C_{S\,e\,p}=h\times \left(H_{1}-h+1\right)+n\times \left(H_{1}-h+1\right)$$

The computational cost of a single-layer standard 1D convolution is expressed as follows:


$$C=n\times h\times \left(H_{1}-h+1\right)$$

The parameter ratio of separable to standard convolution computational costs can be expressed as:


$$\frac{C_{S\,e\,p}}{C}=\frac{1}{n}+\frac{1}{h}$$

Compared with the model of Lawhern et al. (2018), SepCNN had a simpler structure. In addition, SepCNN used a single-layer separable convolutional, which theoretically required less computation than the single-layer standard CNN of Shan et al. (2018). Compared with the CNN proposed by Alvarado-González et al. (2021), SepCNN added more normalization layers for better AR-P300 classification performance.

In this manuscript, SepCNN and standard CNN were used to classify target and non-target stimulus data, which was a binary classification. Next, target stimuli were localized in nine data points generated from one trial to identify the user-selected target, which resulted in nine classification outcomes.

## Results

### Comparison of single extraction results obtained by separable convolutional neural network and classical algorithms

**FIGURE 7:**
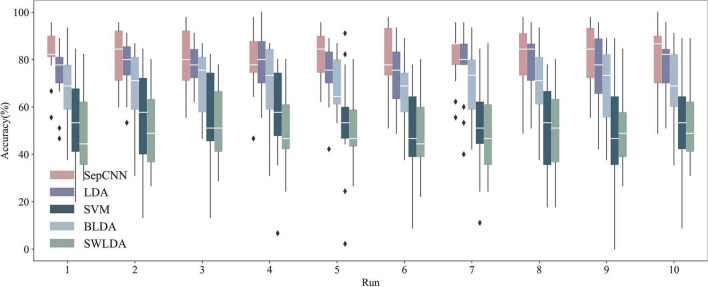
Recognition accuracies of AR–P300 for all participants with different algorithms. The boxes from top to bottom represent the upper quartile (Q1), median (Q2), and lower quartile (Q3), respectively. The values for the upper and lower whiskers are Q1 + 1.5 × (Q1–Q3) and Q3–1.5 × (Q1–Q3), respectively.

The binary classification AUC of SepCNN in AR–P300 single extraction was 0.971, which indicated a good classification performance. In total, nine binary classification results were required to predict one target. The performance of SepCNN in practical applications could not be directly represented by the AUC. The average recognition accuracies of nine targets had to be calculated. Figure 7 shows the within-participant recognition results of all algorithms after five-fold cross-validation. In general, the area between the upper and lower quartiles of SepCNN was higher than that of other methods, indicating that SepCNN had a better recognition performance in single extraction. The overall average accuracy of SepCNN was 81.1%, and participant 6 reached 100% accuracy in run10. The descending order of the classification effects of the five methods was SepCNN > LDA > SVM > BLDA > SWLDA.

**FIGURE 8:**
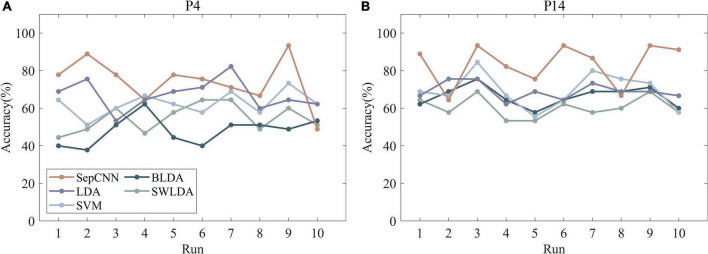
Recognition accuracies of two participants **(A,B)** (participants 4 and 14) with different algorithms.

Figure 8, which presents the recognition accuracies of participants 4 and 14 with different algorithms, shows that SepCNN outperformed other algorithms in most cases.

Table 4 displays the detailed recognition results of SepCNN on AR–P300 offline data. The recognition accuracy of run1 was the highest, and the accuracy decreased as the number of runs increased. The above results were consistent with the reported feelings of the participants. Different degrees of discomfort were expressed by all 15 participants while wearing the Hololens throughout the 40-min experiment. Participant 15 experienced double vision due to their inability to distinguish the depth difference between the stimulus and the background, which led to the lowest recognition accuracy. Even so, the average recognition accuracy of SepCNN in single extraction was 81.1%, which indicated that SepCNN is effective for the single extraction of AR–P300 features.

**TABLE 4:** Results of AR–P300 recognition in SepCNN.

Participant	Accuracy per run (%)										
	1	2	3	4	5	6	7	8	9	10	Mean
P1	91.11	84.44	97.78	97.78	95.56	93.33	86.66	95.55	75.55	86.66	90.44
P2	95.56	93.33	88.89	86.67	95.56	77.78	88.89	88.89	55.56	86.67	85.78
P3	88.89	88.89	95.56	77.78	66.67	97.78	86.67	88.89	93.33	88.89	87.34
P4	77.78	88.89	77.78	64.44	77.77	75.56	71.11	66.67	93.33	48.89	74.22
P5	82.22	84.44	80.00	86.67	88.89	91.11	86.67	82.22	97.78	82.22	86.22
P6	82.22	93.33	71.11	88.89	68.89	77.78	86.67	93.33	71.11	100.0	83.33
P7	82.22	95.56	93.33	91.11	86.67	97.78	95.56	97.78	84.44	97.78	93.33
P8	91.11	95.56	64.44	75.56	95.56	93.33	80.00	86.67	86.67	93.33	86.22
P9	82.22	75.56	71.11	73.33	84.44	88.89	62.22	95.56	86.67	88.89	80.89
P10	88.89	91.11	84.44	75.56	91.11	51.11	86.67	84.44	84.44	68.89	80.67
P11	91.11	75.56	91.11	95.56	80.00	71.11	82.22	82.22	73.33	66.67	80.89
P12	55.56	66.67	80.00	46.67	62.22	71.11	82.22	68.89	62.22	71.11	66.67
P13	80.00	62.22	60.00	77.78	73.33	75.56	75.56	77.78	93.33	60.00	73.56
P14	88.89	64.44	93.33	82.22	75.56	93.33	86.67	66.67	93.33	91.11	83.56
P15	66.67	60.00	55.56	64.44	84.44	60.00	55.56	48.89	66.67	71.11	63.33
**Mean**	82.97	81.37	80.29	78.97	81.79	81.04	80.91	81.64	81.18	80.15	81.10

### Comparison results of separable convolutional neural network in single extraction and classic algorithm in multiple averaging

**FIGURE 9:**
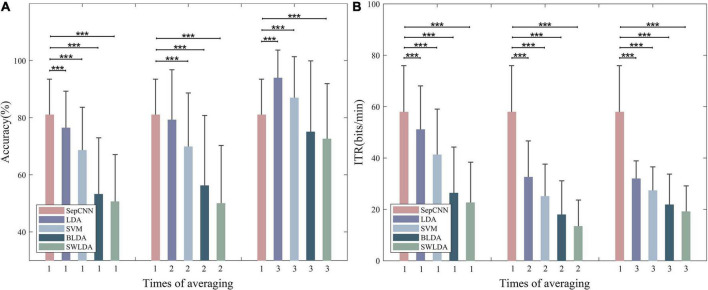
**(A)** Recognition accuracy and **(B)** ITR of SepCNN with single extraction and four classical algorithms with single extraction and multiple averaging. *p < 0.05, **p < 0.01, ***p < 0.001.

The single extraction was defined as classification using EEG data of only once trial. Figure 9 compares the recognition (see Figure 9A) and ITR (see Figure 9B) results of SepCNN with single extraction and the four classical algorithms with single extraction and multiple averaging. The results of the paired *t*-test for the recognition accuracy showed that, with single extraction, SepCNN had an extremely significant improvement compared with all classical algorithms [LDA: 76.34% (*p* < 0.001, *t* = 4.72), SVM: 68.58% (*p* < 0.001, *t* = 7.91), BLDA: 53.23% (*p* < 0.001, *t* = 14.55), SWLDA: 50.62% (*p* < 0.001, *t* = 18.44)]. When the classical algorithms were averaged twice, SepCNN still had an extremely significant improvement in accuracy (*p* < 0.001) compared with SVM [69.85% (*t* = 7.29)], BLDA [56.22% (*t* = 12.74)], and SWLDA [50% (*t* = 17.32)], but it did not have a significant improvement (*p* > 0.05) compared with LDA [79.2% (*t* = 1.20)]. When the classical algorithms were averaged three times, the recognition accuracy of SepCNN was significantly low (*p* < 0.001) compared with LDA [93.93% (*t* = −11.60)] and SVM [86.96% (*t* = −4.38)], high compared with BLDA [75.04% (*t* = −3.14)], and extremely significantly high compared with SWLDA [72.59% (*t* = 5.00)].

Increasing the number of times of averaging did not improve the ITR of the classical algorithms. The paired *t*-test results showed that the ITR (57.90 bits/min) of SepCNN was extremely significantly higher than other classical algorithms for different numbers of averaging. When the average number of times was one, the *t*-test results were as follows: LDA (*p* < 0.001, *t* = 4.38), SVM (*p* < 0.001, *t* = 9.94), BLDA (*p* < 0.001, *t* = 18.80), SWLDA (*p* < 0.001, *t* = 21.25). In the AR–P300 paradigm, the system response time was divided into stimulation time, wireless transmission delay, and data interception delay.

When the classical algorithms were averaged more than twice, the advantage of SepCNN with single extraction was no longer obvious. However, SepCNN required the least recognition time. In summary, SepCNN has significant advantages in the construction of a fast AR–P300 system.

### Performance of separable convolutional neural network in different experimental environments

**FIGURE 10:**
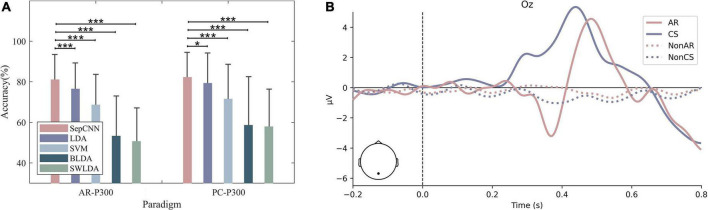
**(A)** Recognition accuracy and **(B)** average amplitude at Oz of all participants for CS– P300 and AR–P300. *p < 0.05, **p < 0.01, ***p < 0.001.

Figure 10A shows the comparison of the average recognition accuracies of the single extraction of SepCNN in CS–P300 and AR–P300. The results of the paired *t*-test for the CS–P300 showed that, with single extraction, the accuracy of SepCNN (82.24%) was significantly higher than LDA [79.25% (*p* < 0.05, *t* = 2.30)], extremely significantly higher than SVM [71.47% (*p* < 0.001, *t* = 7.32)], BLDA [58.56 % (*p* < 0.001, *t* = 12.34)] and SWLDA [57.87% (*p* < 0.001, *t* = 15.34)]. The overall accuracy of CS–P300 was higher than that of AR–P300 because the AR–P300 experiment had many interference factors. In addition, the accuracy of the same algorithm in different environments was further compared. The paired *t*-test results showed that, SepCNN was not significantly different (*p* > 0.05, *t* = 1.10), LDA (*p* < 0.05, *t* = 2.40) and SVM (*p* < 0.05, *t* = 2.15) were significantly different, there was an extremely significant difference in BLDA (*p* < 0.001, *t* = 3.76) and SWLDA (*p* > 0.001, *t* = 7.10). The above results show that, compared with the CS–P300 experiment, SepCNN has a greater improving effect on the accuracy of AR–P300.

A further waveform comparison of the two paradigms indicated that there were differences in both amplitude and delay. Figure 10B shows the waveforms at Oz in two experimental environments, where solid and dashed lines represent target and non-target stimuli, respectively. The amplitude of AR–P300 was lower than that of CS–P300. Table 5 shows the average latency of P300 for 15 participants in both environments, AR–P300 is about 47 ms longer than CS–P300.

**TABLE 5:** Average latency of P300 for all participants.

Participant	PC (ms)	AR (ms)
P1	427	478
P2	434	477
P3	437	486
P4	437	487
P5	438	483
P6	436	484
P7	435	482
P8	431	479
P9	431	481
P10	431	481
P11	433	481
P12	436	482
P13	438	485
P14	438	485
P15	436	486
Mean	434.53	482.47

In summary, the P300 amplitudes and latencies of the two environments were significantly different but the accuracy rates were not, indicating that the recognition results of SepCNN may were less affected by the experimental environment.

## Discussion

### Performance influencing factors of separable convolutional neural network in single extraction

This study attempted to consider multiple participants at a time (cross-participant) for training. Specifically, 80% of the participants were used for training, and the rest were used for testing. However, the AR–P300 average recognition result was less than 50% after five-fold cross-validation. The reason may be that SepCNN had poor performance in identifying AR–P300 signals with large individual differences.

**FIGURE 11:**
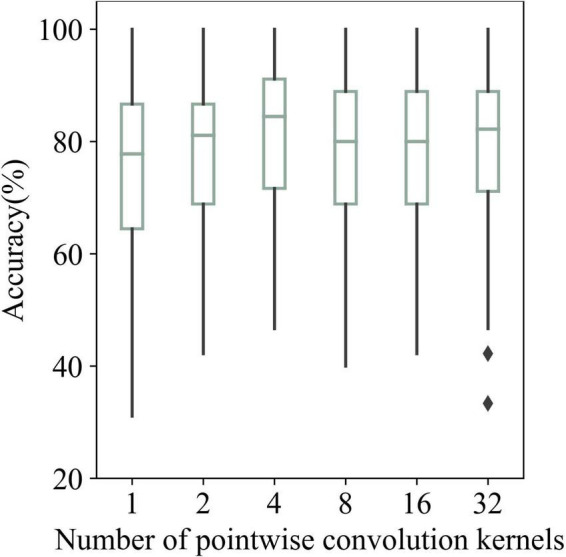
Recognition accuracies of AR–P300 for all participants with different numbers of pointwise convolution kernels.

Figure 11 shows the average recognition results of AR–P300 by SepCNN with different numbers of pointwise convolution kernels. The recognition results for 1, 2, 4, 8, 16, and 32 kernels were 74.82, 78, 81.10, 78.28, 77.97, and 78.43%, respectively.

**FIGURE 12:**
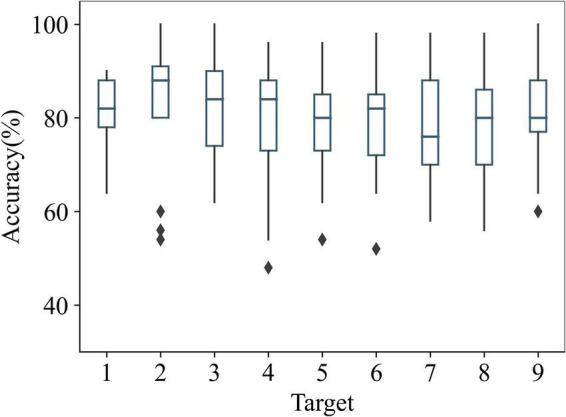
Recognition accuracies of AR–P300 for all participants with different targets.

Previous studies (Krusienski et al., 2006; Zhao et al., 2020) showed that the recognition rate was lower for stimuli at the edges of AR lenses. Figure 12 shows the recognition accuracies of AR–P300 for all participants with different targets. Target 2 had the highest recognition accuracy (82.8%), and target 7 had the lowest recognition accuracy (78.13%). This aligns with the findings of the previous studies since target 7 was closer to the edge of the lens than target 2. One-way ANOVA showed that stimulus location had no significant effect on accuracy (*F*(8,126) = 0.323, *p* > 0.05). In addition, according to paired *t*-test, the recognition accuracy of target 7 was not significantly different from the remaining eight targets (*p* > 0.05). This shows that the recognition performance of SepCNN was not significantly affected by the stimulus location in the AR–P300 paradigm.

### Performance comparison of separable convolutional neural network and standard convolutional neural network in single extraction

For AR–P300 single-experiment recognition accuracy, SepCNN (81.1%) was higher than standard CNN (78%), but there was no significant difference (*p* > 0.05, *t* = 1.89); standard CNN was significantly higher than LDA algorithm [*p* < 0.05, *t* = 2.39)], while SepCNN was extremely significantly higher than LDA [*p* < 0.001, *t* = 4.72)]. The above results show that, compared with the classical algorithm, the recognition accuracy improvements of SepCNN for AR–P300 is greater than that of standard CNN. Table 6 shows the recognition times of the two CNNs for 90 AR–P300 targets under different computing environments.

**TABLE 6:** Recognition times of the two CNNs for 90 AR–P300 targets.

Environment	Time for different models (ms)		
	CNN	Sep–CNN	Difference
AR	66.656	50.137	16.519
CS	11.832	10.774	1.058

The recognition time gap between the two CNNs was larger in the AR environment (16.519 ms) than in the CS environment (1.058 ms). The reason may be that the computing power of the AR device was inferior to that of the CS, and the computational cost of SepCNN was less. The above results show that both the recognition and deployment performance of SepCNN was better than standard CNN in single extraction in AR–P300. When the input data or application scenarios are complex, such as the multi-person collaboration of AR–P300, the impact of algorithm classification delay will increase on the response speed of the system. At this time, the advantages of SepCNN will be more obvious.

## Conclusion

This study evaluated the effectiveness of a lightweight CNN based on separable convolutions for AR–P300 single extraction. Compared with the classical machine learning algorithms, SepCNN significantly improved the ITR and recognition accuracy; compared with standard single-layer CNN, SepCNN had low complexity and significantly improved the computational efficiency deployed in an AR wearable device. Although the recognition performance of SepCNN in the simple AR–P300 was generally ideal, as the stimulus environment becomes more complex, the non-target stimulus and environment interfaces will increase. Moreover, the overall performance of SepCNN will drop dramatically as the command set increases. In the future, we will study the performance of deep learning algorithms in single extraction in complex AR–P300 environments. In addition, we will try to design AR–based P300 and SSVEP hybrid BCI to further improve the practicability of AR–BCI.

## Data availability statement

The raw data supporting the conclusions of this article will be made available by the authors, without undue reservation.

## Ethics statement

The protocol of the experiment was designed according to the Declaration of Helsinki (except for preregistration) and approved by the University of Zhengzhou Ethics Committee (Ethics Review Form Number: ZZUIRB2021-92). The patients/participants provided their written informed consent to participate in this study.

## Author contributions

CH: data curation, methodology, and writing – original draft. XZ: funding acquisition and project administration. CH and YD: software and writing – review and editing. All authors contributed to the article and approved the submitted version.

## References

[B1] Alvarado-González M. Pineda G. F. Cervantes-Ojeda J. (2021). A few filters are enough: Convolutional neural network for P30detection. *Neurocomputing* 425 37–52. 10.1016/j.neucom.2020.10.104

[B2] Bostanov V. (2004). BCI competition 2003-data sets Ib and IIb: Feature extraction from event-related brain potentials with the continuous wavelet transform and the t-value scalogram. *IEEE Trans. BioMed. Eng.* 51 1057–1061. 10.1109/TBME.2004.826702 15188878

[B3] Cecotti H. Graser A. (2011). Convolutional neural networks for P30detection with application to Brain-Computer Interfaces. *IEEE Trans. Pattern. Anal.* 33 433–445. 10.1109/TPAMI.2010.125 20567055

[B4] Chollet F. (2017). “Xception: Deep learning with depthwise separable convolutions,” in *Proceedings of the 2017 IEEE Conference on Computer Vision and Pattern Recognition (CVPR)*, (Honolulu: IEEE). 10.1109/CVPR.2017.195

[B5] Farwell L. A. Donchin E. (1988). Talking off the top of your head: Toward a mental prosthesis utilizing event-related brain potentials. *Electroecephalogr. Clin. Neurophysiol.* 70 510–523. 10.1016/0013-4694(88)90149-510-23 2461285

[B6] Guger C. Daban S. Sellers E. Holzner C. Krausz G. Carabalona R. (2009). How many people are able to control a p300-based brain-computer interface (bci)? *Neurosci. Lett.* 462 94–98. 10.1016/j.neulet.2009.06.045 19545601

[B7] Hoffmann U. Vesin J. M. Ebrahimi T. Diserens K. (2008). An efficient P300-based brain-computer interface for disabled subjects. *J. Neurosci. Meth.* 167 115–125. 10.1016/j.jneumeth.2007.03.005 17445904

[B8] Jin J. Sellers E. W. Wang X. (2012). Targeting an efficient target-to-target interval for p30speller brain–computer interfaces. *Med. Biol. Eng. Comput.* 50 289–296. 10.1007/s11517-012-0868-x 22350331 PMC3646326

[B9] Kaper M. Meinicke P. Grossekathoefer U. Lingner T. Ritter H. (2004). BCI competition 2003-data set IIb: Support Vector Machines for the P30speller paradigm. *IEEE Trans. BioMed. Eng.* 51 1073–1076. 10.1109/TBME.2004.826698 15188881

[B10] Kerous B. Liarokapis F. (2017). “BrainChat—A collaborative augmented reality brain interface for message communication,” in *Proceedings of the 2017 IEEE International Symposium on Mixed and Augmented Reality (ISMAR-Adjunct)*, (Nantes: IEEE), 279–283. 10.1109/ISMAR-Adjunct.2017.91

[B11] Kim S. Lee S. Kang H. Kim S. Ahn M. (2021). P30Brain–computer interface-based drone control in virtual and augmented reality. *Sensors* 21:5765. 10.3390/s21175765 34502655 PMC8434009

[B12] Kouji T. Hata N. Kansaku K. (2011). Towards intelligent environments: An augmented reality–brain–machine interface operated with a see-through head-mount display. *Front. Neurosci.* 5:60. 10.3389/fnins.2011.00060 21541307 PMC3082767

[B13] Krusienski D. J. Sellers E. W. Cabestaing F. Bayoudh S. McFarland D. J. Vaughan T. M. (2006). A comparison of classification techniques for the P30speller. *J. Neural. Eng.* 3 299–305. 10.1088/1741-2560/3/4/007 17124334

[B14] Lawhern V. J. Solon A. J. Waytowich N. R. Gordon S. M. Hung C. P. Lance B. J. (2018). EEGNet: A compact convolutional neural network for EEG-based brain-computer interfaces. *Neural. Eng.* 15:056013. 10.1088/1741-2552/aace8c 29932424

[B15] Lenhardt A. Ritter H. (2010). “An augmented-reality based brain-computer interface for robotcontrol,” in *Proceedings of the International Conference on Neural Information Processing*, (Berlin: Springer). 10.1007/978-3-642-17534-3_8

[B16] Manor R. Geva A. B. (2015). Convolutional neural network for multi-category rapid serial visual presentation BCI. *Front. Comput. Neurosci.* 9:146. 10.3389/fncom.2015.00146 26696875 PMC4667102

[B17] Nijboer F. Sellers E. W. Mellinger J. Jordan M. A. Matuz T. Furdea A. (2008). A P300-based brain-computer interface for people with amyotrophic lateral sclerosis. *Clin. Neurophysiol.* 119 1909–1916. 10.1016/j.clinph.2008.03.034 18571984 PMC2853977

[B18] Rohani D. A. Puthusserypady S. (2015). BCI inside a virtual reality classroom: A potential training tool for attention. *EPJ Nonlinear. Biomed. Phys.* 3 1–14. 10.1140/epjnbp/s40366-015-0027-z

[B19] Sellers E. W. Donchin E. (2006). A P300-based brain-computer interface: Initial tests by ALS patients. *Clin. Neurophysiol.* 117 538–548. 10.1016/j.clinph.2005.06.027 16461003

[B20] Sellers E. W. Krusienski D. J. McFarland D. J. Vaughan T. M. Wolpaw J. R. (2006). A p30event-related potential braincomputer interface (bci): The effects of matrix size inter stimulus interval on performance. *Biol. Psychol.* 73 242–252. 10.1016/j.biopsycho.2006.04.007 16860920

[B21] Shan H.C. Liu Y. Stefanov T. (2018). A simple convolutional neural network for accurate P30detection and character spelling in brain computer interface. Vienna: IJCAI, 1604–1610. 10.24963/ijcai.2018/222

[B22] Si-Mohammed H. Petit J. Jeunet C. Spindler F. Evain A. Argelaguet F. (2018). Towards bci-based interfaces for augmented reality: Feasibility, design and evaluation. *IEEE Trans. Vis. Comput. Graph.* 26 1608–1621. 10.1109/TVCG.2018.2873737 30295623

[B23] Vapnik V. (1998). *Statistical Learning Theory.* Saarland: DBLP.

[B24] Xu M. He F. Jung T. P. Gu X. Ming D. (2021). Current challenges for the practical application of electroencephalography-based brain–computer interfaces. *Engineering* 7:3. 10.1016/j.eng.2021.09.01133520329

[B25] Zhao X. Du Y. Zhang R. (2021). A cnn-based multi-target fast classification method for ar-ssvep. *Comput. Biol. Med.* 141:105042. 10.1016/j.compbiomed.2021.105042 34802710

[B26] Zhao X. Liu C. Xu Z. Zhang L. Zhang R. (2020). SSVEP Stimulus layout effect on accuracy of brain-computer interfaces in augmented reality glasses. *IEEE Access.* 8 5990–5998. 10.1109/ACCESS.2019.2963442

